# Results of the cadaveric kidney transplantations according to national data of turkish organ and tissue information system

**DOI:** 10.1186/2197-425X-3-S1-A902

**Published:** 2015-10-01

**Authors:** Ç Kaymak, İ Şencan, A Kapuağası, MA Aydın, M Öztürk, Z Uzundurukan, AH Elhan, A Özcan, H Başar

**Affiliations:** Ankara Training and Research Hospital, Ministry of Health, Department of Anesthesiology and Reanimation, Ankara, Turkey; General Directorate of Health Services, Ministry of Health, Ankara, Turkey; General Directorate of Health Services, Ministry of Health, Organ, Tissue and Transplantation Department, Ankara, Turkey; Faculty of Medicine, Ankara University, Department of Biostatistics, Ankara, Turkey

## Introduction

Cadaveric kidney transplantation is performed according to a patient list formed by matching and point score in our country. Demand for renal transplantation can not be met by the number of cadavers introduced to the system because of the increase in number of patients with chronic renal failure who require renal transplantation.

## Objectives

We reviewed the results of the patients who had cadaveric kidney transplantation to assess the kidney transplantations in the Turkish Organ and Tissue Information System.

## Methods

The results of the patients who had cadaveric kidney transplantation between years 2011-2014 were reviewed. Patients' age, gender and tissue antigen integration were determined. The chronic rejection and primary graft failure rates were recorded. Survival rates of the grafts and patients during 3, 6, 9, 12, 24, 36 ve 48 months were determined. Kaplan-Meier method was used to calculate the survival rates.

## Results

The number of kidney transplantations was 11755 between years 2011-2014 in Turkey. The source of organ in 19.2 % of the transplantations was cadaveric. The mean age of the patients who had cadaveric kidney transplantation was 42.3 ± 14.4 (mean ± SD), and 57.6% (1301) of the patients were male and 42.4% (957) were female. Chronic rejection and primary graft failure were determined in 4.9% (110) of the patients. Patient and graft follow-up periods were 23.4 ± 14.9 (mean ± SD) months and 22.7 ± 14.4 (mean ± SD) months, respectively. Mean survival time of the patients was 46.81 ± 0.288 months, and survival times for 3, 6, 9, 12, 24, 36 and 48 months were % 95.3 ± 0.004; % 93.4 ± 0.005; % 92.4 ± 0.006; %91.8 ± 0.006; % 91.4 ± 0.006; %91.3 ± 0.006 and % 91.1 ± 0.006, respectively. Mean survival time of the graft was 46.56 ± 0.23 months in these patients and survival times of the graft for 3, 6, 9, 12, 24, 36 and 48 months were % 98.1 ± 0.003; %97.2 ± 0.004; %96.3 ± 0.004; %96.4 ± 0.004; %95 ± 0.005; %94.1 ± 0.006 and %91.7 ± 0.013, respectively.

## Conclusions

Although there are attempts to increase cadaveric donation in the whole world, it is still poor because of the reluctance and irrelevance of the donor families. Even though, the patient and graft survival rates were higher in live kidney transplantations than cadaveric kidney transplantations. In recent decade, cadaver-kidney transplantations also had high success rates. In our opinion, cadaver-kidney transplantations should be increased in respect of the results of the cadaver-kidney transplantations in our country.
